# {4,4′-Dibromo-2,2′-[propane-1,3-diyl­bis(nitrilo­methyl­idyne)]diphenolato}zinc(II)

**DOI:** 10.1107/S1600536808015602

**Published:** 2008-05-30

**Authors:** Jun-Ying Ma

**Affiliations:** a Chemical Engineering and Pharmaceutics College, Henan University of Science and Technology, Luoyang, Henan 471003, People’s Republic of China, and Department of Chemistry, Pingdingshan University, Pingdingshan, Henan 467000, People’s Republic of China

## Abstract

The title mononuclear zinc(II) complex, [Zn(C_17_H_14_Br_2_N_2_O_2_)], possesses a crystallographically imposed *C*_2_ axis. The Zn atom is four-coordinated by two O and two N atoms from two Schiff base ligands, forming a severely distorted square-planar geometry. The central C atom of the propyl group is disordered over two positions about the twofold axis.

## Related literature

For background on the chemistry of Schiff base zinc(II) complexes and their biological activity, see: Anderson *et al.* (1997 ▶); Chohan & Kausar (1992 ▶, 1993 ▶); Chohan *et al.* (2003 ▶); Osowole *et al.* (2005 ▶); Yu *et al.*, (2007 ▶). For related structures, see: Li & Zhang (2005 ▶); Wu *et al.* (2006 ▶); Xu *et al.* (2006 ▶); Ma *et al.* (2005 ▶); Ma, Gu *et al.* (2006 ▶); Ma, Lv *et al.* (2006 ▶); Ma, Wu *et al.* (2006 ▶).
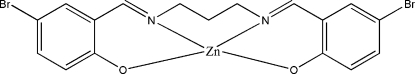


## Experimental

### 

#### Crystal data

[Zn(C_17_H_14_Br_2_N_2_O_2_)]
*M* *_r_* = 503.49
Monoclinic, 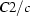

*a* = 21.418 (6) Å
*b* = 8.161 (2) Å
*c* = 9.524 (3) Å
β = 92.910 (3)°
*V* = 1662.6 (8) Å^3^
*Z* = 4
Mo *K*α radiation
μ = 6.30 mm^−1^
*T* = 298 (2) K
0.32 × 0.30 × 0.30 mm

#### Data collection

Bruker SMART CCD area-detector diffractometer
Absorption correction: multi-scan (*SADABS*; Bruker, 2000 ▶) *T*_min_ = 0.143, *T*_max_ = 0.152
4709 measured reflections
1809 independent reflections
1444 reflections with *I* > 2σ(*I*)
*R*_int_ = 0.036

#### Refinement

*R*[*F* ^2^ > 2σ(*F* ^2^)] = 0.036
*wR*(*F* ^2^) = 0.092
*S* = 1.05
1809 reflections
121 parameters
3 restraints
H atoms treated by a mixture of independent and constrained refinement
Δρ_max_ = 0.45 e Å^−3^
Δρ_min_ = −0.64 e Å^−3^

### 

Data collection: *SMART* (Bruker, 2000 ▶); cell refinement: *SAINT* (Bruker, 2000 ▶); data reduction: *SAINT*; program(s) used to solve structure: *SHELXTL* (Sheldrick, 2008 ▶); program(s) used to refine structure: *SHELXTL*; molecular graphics: *SHELXTL*; software used to prepare material for publication: *SHELXTL*.

## Supplementary Material

Crystal structure: contains datablocks global, I. DOI: 10.1107/S1600536808015602/rz2218sup1.cif

Structure factors: contains datablocks I. DOI: 10.1107/S1600536808015602/rz2218Isup2.hkl

Additional supplementary materials:  crystallographic information; 3D view; checkCIF report

## Figures and Tables

**Table:** 

Zn1—O1	1.912 (3)
Zn1—N1	1.968 (3)

**Table:** 

O1—Zn1—O1^i^	87.42 (16)
O1—Zn1—N1^i^	153.75 (12)
O1—Zn1—N1	93.21 (12)

Symmetry code: (i) 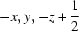.

**Fig. 1.**
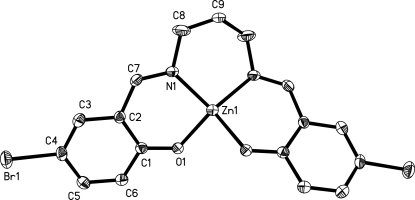
The molecular structure of the title compound with displacement ellipsoids drawn at the 30% probability level. Only one component of the disordered C9 atom is shown. H atoms are omitted for clarity. Unlabelled atoms are related to the labelled atoms by the symmetry operation (-x, y, 1/2-z).

## supplementary crystallographic information

### Comment

Zinc(II) complexes derived from Schiff bases have been widely studied (Anderson *et al.*, 1997). Some of them have been found to have pharmacological and antitumor properties (Chohan & Kausar, 1992, 1993; Osowole *et al.*, 2005; Chohan *et al.*, 2003; Yu *et al.*, 2007). Recently, we have reported some metal complexes derived from the Schiff base ligands (Ma, Lv *et al.*, 2006; Ma, Gu *et al.*, 2006; Ma, We *et al.*, 2005, 2006). As part of a further investigation of the structures of such complexes, the title mononuclear zinc(II) complex (Fig 1) is reported in this paper.

The title compound possesses a crystallographically imposed *C*_2_ axis passing through the zinc(II) atom and the midpoint of the propyl group, causing the C9 atom to be disordered over two positions. The Zn atom is coordinated by two nitrogen atoms and two oxygen atoms from a Schiff base ligand, giving a severely distorted square planar geometry. Bond lengths and angles (Table 1) related to the Zn atom in the complex are within normal ranges, and comparable to the values observed in other Schiff base zinc(II) complexes (Li & Zhang, 2005; Xu *et al.*, 2006; Wu *et al.*, 2006).

### Experimental

*N*,*N*'-Propane-1,3-diamine (0.1 mmol, 14.8 mg) and 5-bromosalicylaldehyde (0.1 mmol, 20.1 mg) were dissolved in methanol (20 ml). The mixture was stirred for 1 h to obtain a clear yellow solution. To the solution was added with stirring a methanol solution (20 ml) of zinc(II) acetate (0.1 mmol, 22.0 mg). After keeping the resulting solution in air for a few days, colourless block-shaped crystals were formed on slow evaporation of the solvent.

### Refinement

H9A and H9B were located from a difference Fourier map and refined freely, with C–H and H···H distances restrained to 0.96 (1) and 1.50 (2) respectively, and with an isotropic displacement parameter fixed to 0.08 Å^2^. %A. Other H atoms were positioned geometrically and refined using a riding model with C—H = 0.93-96 Å and *U*_iso_ = 1.2 *U*_eq_(C).

### Crystal data

**Table:**

[Zn(C_17_H_14_Br_2_N_2_O_2_)]	*F*_000_ = 984
*M**_r_* = 503.49	*D*_x_ = 2.011 Mg m^−^^3^
Monoclinic, *C*2/*c*	Mo *K*α radiation λ = 0.71073 Å
Hall symbol: -C 2yc	Cell parameters from 1595 reflections
*a* = 21.418 (6) Å	θ = 2.5–26.3º
*b* = 8.161 (2) Å	µ = 6.30 mm^−^^1^
*c* = 9.524 (3) Å	*T* = 298 (2) K
β = 92.910 (3)º	Block, colourless
*V* = 1662.6 (8) Å^3^	0.32 × 0.30 × 0.30 mm
*Z* = 4	

### Data collection

**Table:**

Bruker SMART CCD area-detector diffractometer	1809 independent reflections
Radiation source: fine-focus sealed tube	1444 reflections with *I* > 2σ(*I*)
Monochromator: graphite	*R*_int_ = 0.036
*T* = 298(2) K	θ_max_ = 27.0º
ω scans	θ_min_ = 1.9º
Absorption correction: multi-scan(SADABS; Bruker, 2000)	*h* = −27→27
*T*_min_ = 0.143, *T*_max_ = 0.152	*k* = −10→9
4709 measured reflections	*l* = −12→11

### Refinement

**Table:**

Refinement on *F*^2^	Secondary atom site location: difference Fourier map
Least-squares matrix: full	Hydrogen site location: inferred from neighbouring sites
*R*[*F*^2^ > 2σ(*F*^2^)] = 0.036	H atoms treated by a mixture of independent and constrained refinement
*wR*(*F*^2^) = 0.092	*w* = 1/[σ^2^(*F*_o_^2^) + (0.0396*P*)^2^ + 3.1273*P*] where *P* = (*F*_o_^2^ + 2*F*_c_^2^)/3
*S* = 1.05	(Δ/σ)_max_ < 0.001
1809 reflections	Δρ_max_ = 0.45 e Å^−^^3^
121 parameters	Δρ_min_ = −0.64 e Å^−^^3^
3 restraints	Extinction correction: SHELXTL (Bruker, 2000), Fc^*^=kFc[1+0.001xFc^2^λ^3^/sin(2θ)]^-1/4^
Primary atom site location: structure-invariant direct methods	Extinction coefficient: 0.0078 (5)

### Special details

**Table:**

Geometry. All e.s.d.'s (except the e.s.d. in the dihedral angle between two l.s. planes) are estimated using the full covariance matrix. The cell e.s.d.'s are taken into account individually in the estimation of e.s.d.'s in distances, angles and torsion angles; correlations between e.s.d.'s in cell parameters are only used when they are defined by crystal symmetry. An approximate (isotropic) treatment of cell e.s.d.'s is used for estimating e.s.d.'s involving l.s. planes.
Refinement. Refinement of *F*^2^ against ALL reflections. The weighted *R*-factor *wR* and goodness of fit *S* are based on *F*^2^, conventional *R*-factors *R* are based on *F*, with *F* set to zero for negative *F*^2^. The threshold expression of *F*^2^ > σ(*F*^2^) is used only for calculating *R*-factors(gt) *etc*. and is not relevant to the choice of reflections for refinement. *R*-factors based on *F*^2^ are statistically about twice as large as those based on *F*, and *R*- factors based on ALL data will be even larger.

### Fractional atomic coordinates and isotropic or equivalent isotropic displacement parameters (Å^2^)

**Table:**

	*x*	*y*	*z*	*U*_iso_*/*U*_eq_	Occ. (<1)
Zn1	0.0000	0.01735 (7)	0.2500	0.0347 (2)	
Br1	0.23023 (2)	0.25397 (6)	0.81463 (5)	0.0539 (2)	
N1	0.04072 (14)	−0.1416 (3)	0.3803 (3)	0.0343 (7)	
O1	0.05877 (12)	0.1867 (3)	0.2995 (3)	0.0370 (6)	
C1	0.09920 (16)	0.1906 (4)	0.4060 (4)	0.0310 (8)	
C2	0.10887 (15)	0.0587 (4)	0.5015 (4)	0.0298 (7)	
C3	0.14983 (16)	0.0782 (5)	0.6219 (4)	0.0362 (8)	
H3	0.1557	−0.0080	0.6851	0.043*	
C4	0.18070 (17)	0.2229 (5)	0.6459 (4)	0.0367 (8)	
C5	0.17583 (18)	0.3491 (5)	0.5466 (4)	0.0406 (9)	
H5	0.1995	0.4439	0.5598	0.049*	
C6	0.13625 (17)	0.3329 (5)	0.4299 (4)	0.0376 (8)	
H6	0.1336	0.4176	0.3645	0.045*	
C7	0.08158 (16)	−0.1003 (4)	0.4779 (4)	0.0332 (8)	
H7	0.0949	−0.1827	0.5399	0.040*	
C8	0.0244 (3)	−0.3170 (5)	0.3736 (5)	0.0577 (13)	
H8A	0.0119	−0.3481	0.4652	0.069*	
H8B	0.0623	−0.3752	0.3578	0.069*	
C9	−0.0202 (3)	−0.3766 (8)	0.2766 (9)	0.0375 (17)	0.50
H9A	−0.024 (4)	−0.4935 (16)	0.280 (12)	0.080*	0.50
H9B	−0.059 (2)	−0.340 (9)	0.310 (11)	0.080*	0.50

### Atomic displacement parameters (Å^2^)

**Table:**

	*U*^11^	*U*^22^	*U*^33^	*U*^12^	*U*^13^	*U*^23^
Zn1	0.0395 (4)	0.0264 (3)	0.0374 (4)	0.000	−0.0056 (2)	0.000
Br1	0.0500 (3)	0.0645 (4)	0.0453 (3)	−0.0064 (2)	−0.01642 (19)	−0.0038 (2)
N1	0.0442 (17)	0.0228 (15)	0.0354 (17)	0.0001 (13)	−0.0019 (14)	0.0015 (13)
O1	0.0465 (15)	0.0277 (13)	0.0354 (15)	−0.0030 (11)	−0.0111 (11)	0.0031 (11)
C1	0.0307 (18)	0.0281 (18)	0.034 (2)	0.0026 (14)	0.0015 (14)	−0.0022 (14)
C2	0.0269 (16)	0.0318 (19)	0.0304 (18)	0.0034 (14)	−0.0006 (13)	−0.0008 (14)
C3	0.0334 (18)	0.041 (2)	0.034 (2)	0.0074 (16)	0.0008 (15)	0.0032 (16)
C4	0.0270 (18)	0.045 (2)	0.037 (2)	0.0001 (15)	−0.0064 (15)	−0.0041 (17)
C5	0.039 (2)	0.036 (2)	0.046 (2)	−0.0077 (16)	−0.0026 (17)	−0.0016 (18)
C6	0.040 (2)	0.0304 (19)	0.042 (2)	−0.0015 (16)	−0.0026 (16)	0.0028 (16)
C7	0.0371 (19)	0.0305 (19)	0.0317 (19)	0.0077 (15)	−0.0009 (15)	0.0041 (15)
C8	0.097 (4)	0.025 (2)	0.051 (3)	−0.009 (2)	0.000 (3)	0.0003 (19)
C9	0.045 (5)	0.020 (3)	0.047 (5)	−0.001 (3)	−0.003 (4)	0.005 (3)

### Geometric parameters (Å, °)

**Table:**

Zn1—O1	1.912 (3)	C4—C5	1.398 (6)
Zn1—O1^i^	1.912 (3)	C5—C6	1.370 (5)
Zn1—N1^i^	1.968 (3)	C5—H5	0.9300
Zn1—N1	1.968 (3)	C6—H6	0.9300
Br1—C4	1.897 (4)	C7—H7	0.9300
N1—C7	1.289 (5)	C8—C9	1.383 (9)
N1—C8	1.474 (5)	C8—C9^i^	1.509 (9)
O1—C1	1.301 (4)	C8—H8A	0.9600
C1—C2	1.417 (5)	C8—H8B	0.9599
C1—C6	1.419 (5)	C9—C9^i^	1.025 (15)
C2—C3	1.417 (5)	C9—C8^i^	1.509 (9)
C2—C7	1.436 (5)	C9—H9A	0.958 (10)
C3—C4	1.367 (5)	C9—H9B	0.960 (10)
C3—H3	0.9300		
			
O1—Zn1—O1^i^	87.42 (16)	C5—C6—C1	121.8 (4)
O1—Zn1—N1^i^	153.75 (12)	C5—C6—H6	119.1
O1^i^—Zn1—N1^i^	93.21 (12)	C1—C6—H6	119.1
O1—Zn1—N1	93.21 (12)	N1—C7—C2	127.3 (3)
O1^i^—Zn1—N1	153.75 (12)	N1—C7—H7	116.4
N1^i^—Zn1—N1	97.53 (18)	C2—C7—H7	116.4
C7—N1—C8	115.9 (3)	C9—C8—N1	121.7 (5)
C7—N1—Zn1	123.0 (2)	N1—C8—C9^i^	110.9 (4)
C8—N1—Zn1	121.1 (3)	C9—C8—H8A	107.4
C1—O1—Zn1	127.9 (2)	N1—C8—H8A	107.1
O1—C1—C2	123.5 (3)	C9^i^—C8—H8A	140.4
O1—C1—C6	119.3 (3)	C9—C8—H8B	106.4
C2—C1—C6	117.2 (3)	N1—C8—H8B	106.6
C1—C2—C3	119.9 (3)	C9^i^—C8—H8B	72.7
C1—C2—C7	122.7 (3)	H8A—C8—H8B	106.8
C3—C2—C7	117.3 (3)	C9^i^—C9—C8	76.0 (9)
C4—C3—C2	120.4 (3)	C9^i^—C9—C8^i^	62.8 (8)
C4—C3—H3	119.8	C8—C9—C8^i^	121.7 (6)
C2—C3—H3	119.8	C9^i^—C9—H9A	95 (4)
C3—C4—C5	120.3 (4)	C8—C9—H9A	112 (7)
C3—C4—Br1	120.1 (3)	C8^i^—C9—H9A	111 (7)
C5—C4—Br1	119.6 (3)	C9^i^—C9—H9B	160 (5)
C6—C5—C4	120.0 (3)	C8—C9—H9B	105 (6)
C6—C5—H5	120.0	C8^i^—C9—H9B	102 (6)
C4—C5—H5	120.0	H9A—C9—H9B	103 (2)

Symmetry codes: (i) −*x*, *y*, −*z*+1/2.
